# Ferrocene thiazolidine-2,4-dione derivatives cause DNA damage and interfere with DNA repair in triple-negative breast cancer cells

**DOI:** 10.1371/journal.pone.0328155

**Published:** 2025-07-17

**Authors:** Nyeleti Vukea, Ogunyemi Oderinlo, Matshawandile Tukulula, Setshaba David Khanye, Adrienne Lesley Edkins, Jo-Anne de la Mare

**Affiliations:** 1 Department of Biochemistry, Microbiology and Bioinformatics, Rhodes University, Makhanda (Grahamstown), South Africa; 2 Department of Chemistry, Rhodes University, Makhanda (Grahamstown), South Africa; 3 Department of Chemistry, Federal University, Otuoke, Bayelsa State, Nigeria; 4 School of Chemistry and Physics, College of Agriculture, Engineering and Science, University of KwaZulu-Natal, Durban, South Africa; 5 Division of Pharmaceutical Chemistry, Rhodes University, Makhanda (Grahamstown), South Africa; Kafrelsheikh University Faculty of Pharmacy, EGYPT

## Abstract

In this study, ferrocene-containing thiazolidine-2,4-dione derivatives were screened against triple-negative breast cancer (TNBC) cell lines, which represent an aggressive subtype of the disease predominant in women of African descent. The lack of key receptors in TNBC poses a therapeutic challenge as there are limited targeted treatment options available for this subtype. The ferrocene thiazolidine-2,4-dione derivatives displayed toxicity against HCC70 TNBC cells in the low-moderate micromolar range (5–46 μM) and two compounds were selected for further study, with IC_50_ values of 7.54 ± 1.07 μM (OY25) and 5.59 ± 1.24 μM (OY29). Additionally, compounds OY25 and OY29 were screened against other cancer and non-tumourigenic cell lines and found to be less toxic against non-cancerous breast epithelial cell line MCF-12A (SI = 2.2188 and 4.4359, respectively) compared to the HCC70 TNBC cell line. Compounds OY25 and OY29 show a dual mode of action involving increased reactive oxygen species generation and induction of DNA damage. *In silico* docking analysis and competitive DNA binding fluorescence-based assays revealed that the compounds disrupt key DNA damage phosphoprotein levels through binding to the minor groove of DNA. In a combination assay, the compounds acted synergistically and antagonistically with DNA damage-inducing drugs, camptothecin and etoposide, respectively. Meanwhile, in combination with PARP-1 inhibitor, OY25 and OY29 acted synergistically and antagonistically, respectively. Furthermore*, in silico* results, using the SwissADME web tool, showed that the compounds OY25 and OY29 display desirable ADME (absorption, distribution, metabolism, and excretion) profile with parameters within acceptable range.

## Introduction

Cancer is responsible for significant human mortality around the world [1]. In women, breast cancer is the most prevalent cancer globally and is the leading cause of cancer-related death [2,3]. In fact, worldwide, breast cancer has surpassed lung cancer as the most frequently diagnosed cancer [1,3]. Triple-negative breast cancer (TNBC) [Oestrogen receptor (ER-, ERβ+), progesterone receptor (PR-), and human epidermal growth factor receptor 2 (HER2-), marker of proliferation Kiel 67 (Ki67high)] is described as an aggressive tumour phenotype as it has an earlier onset and is associated with higher mortality rates compared to other subtypes, with high rates of metastasis and recurrence [4–10]. Several studies revealed that this form of the disease is more prevalent in women of African descent [9,11,12]. Regrettably, there are limited treatment options available for TNBC and the therapies targeting the ER (tamoxifen) and HER2 (trastuzumab) are not effective due to the absence of these receptors, resulting in a poor prognosis for these patients [6–8,11,13]. There is thus a continued need for the identification of new chemical entities with activity against TNBC that may be developed into future therapies.

One of the key hallmarks of cancer cells is their ability to enable genome instability [14,15]. Several mutations driving such genomic instability have been identified in TNBC [16], including germline mutations in *BRCA1* and *TP53*, mutated in approximately 60–80% [17,18] and 80% [19–21] of TNBC patients, respectively. These mutations result in the disruption of key DNA repair pathways [22]. Therefore, disruption of the already weakened DNA repair pathways of TNBC, could represent a promising therapeutic target for this subtype of the disease [23]. Poly (ADP-ribose) polymerase (PARP) inhibitors have shown promising anticancer activity in TNBC and other cancer patients carrying BRCA1 or BRCA2 mutations through a process known as synthetic lethality [23]. PARP inhibition, in combination with cytotoxic DNA-damaging therapeutic agents has been shown to impair the DNA repair process [23–26].

One of the most widely studied heterocyclic motifs is thiazolidinedione [27], characterised by a five-membered ring system that contains a sulfur atom at position 1, nitrogen atom at position 3, and two carbonyl groups at positions 2 and 4 [28,29]. Thiazolidinediones are among the most clinically utilized scaffolds and have important biological activities, in particular antitubercular, antimicrobial, anti-inflammatory, antiviral, antitumour, antifungal, antiplasmodial, antiparasitic and anticancer activities [30–37]. On the other hand, the ferrocene scaffold is an important and useful organometallic unit, due to its ease of synthesis, stability, redox activity, structure and rigid backbone [38], making the ferrocene suitable for biological applications [37]. Compounds containing ferrocene have been shown to exhibit various biological activities including antimalarial, anticancer, antitumor, antifungal, antileishmanial, antiplasmodial and antitrypanosomal activities [37,39]. In this study, we sought to examine the *in vitro* anti-tumour activity of ferrocene-containing thiazolidine-2,4-dione derivatives, a class of compounds previously explored for antiparasitic activity, against the strain of *Plasmodium falciparum* and *Trypanosoma brucei brucei* [37]. These ferrocene-containing thiazolidine-2,4-dione derivatives are structurally similar, with the variation only at the carboxamide region of the compounds, where they are coupled with a selection of secondary cyclic amines and the presence of additional electronegative atoms, oxygen and sulfur, other than nitrogen. The presence of oxygen and sulfur enhanced activity in the previous study, suggesting that the hydrophobicity is an important parameter for these compounds, while the addition of another amine group led to the loss of activity [37]. The well-established anti-cancer activity of both the ferrocene and thiazolidinedione moieties and the increased antiparasitic activity upon addition of electronegative atoms led to the hypothesis that the compounds under study would be active against triple-negative breast cancer cell lines.

Herein, we assessed these compounds for cytotoxicity and DNA damage potential in TNBC cells as potential novel therapeutics for this aggressive subtype, along with their DNA binding capabilities evaluated through *in silico* docking analysis and competitive fluorescence-based assays. Pharmacokinetic and pharmacodynamic profiles were also characterized to gain insights into the compounds’ absorption, distribution, metabolism, and excretion (ADME).

## Materials and methods

### Cell lines and culture conditions

The TNBC cell lines, HCC70 (ATCC-CRL-2315) and HCC1937 (ATCC-CRL-223) basal triple-negative breast cancer (TNBC) cell lines were maintained as previously described [40]. The MCF-7 (ATCC-HTB-22) hormone receptor positive breast cancer, HeLa (ATCC-CCL-2) cervical cancer [41] and MEF-1 (ATCC-CRL-2214) mouse embryonic fibroblast cell lines were maintained as previously described [42].

### Cytotoxicity assays

Cell viability upon treatment with the ferrocene-containing thiazolidine-2,4-dione derivatives was assessed using the MTT assay. The cells were seeded at 5 × 10^3^ cells/well overnight and treated with either the thiazolidine-2,4-dione compounds at 0.16–500 µM, a 1% (v/v) DMSO vehicle control, geldanamycin at 0.8–2500 nM or paclitaxel at 0.16–500 nM as positive controls. After treatment for 96 hours, the MTT assay was carried out as previously described [41] with modification. The absorbance was measured at 570 nm, as the previous study used an MTT reagent from a Cell Proliferation kit I from Roche, which allowed a wavelength range of 550–600 nm. In contrast, the MTT reagent in this study was from Sigma Aldrich, where the absorbance peak of the formazan product is specifically measured at 570 nm. The experiment was carried out in technical triplicates and the data was analyzed using GraphPad Prism 4 Inc (USA) with half-maximal inhibitory concentrations (IC_50_ values) determined by non-linear regression. For synergy studies, the HCC70 cells were seeded at 5 × 10^3^ cells/well overnight and treated with either a 1% (v/v) DMSO vehicle control, etoposide at 0.20–625 μM, camptothecin at 0.005–15.625 μM or AZD2461 at 0.16–500 μM for 96 hours. After treatment, the resazurin assay was carried out as previously described [43] with the addition of 0.54 mM resazurin solution. The experiment was carried out in technical triplicates and data was analyzed as described above.

### Alkaline Comet assay

HCC70 cells were seeded at 2 × 10^5^ cells/well in a 24-well plate overnight and treated with 20 µM H_2_O_2_ (1 hour), 0.01% (v/v) DMSO vehicle control, 7.5 µM OY25 or 5.5 µM OY29 for 2 hours. Frosted slides were pre-coated with 1% (w/v) low-gelling temperature agarose; thereafter, cells and 1% (w/v) molten low-gelling temperature agarose were rapidly mixed and placed onto the agarose coated slides. The alkaline cell lysis, electrophoresis and visualization was performed as previously described [40].

### Reactive oxygen species (ROS) detection using 2′,7′-dichlorofluorescein diacetate (DCFDA)

HCC70 cells were seeded at 1 × 10^4^ cells/well in a 96-well plate overnight in 2% (v/v) serum-containing phenol red-free RPMI-1640 medium. Cells were stained with 5 μM DCFDA in phosphate buffered saline (137 mM NaCl; 2.7 mM KCl; 10 mM Na_2_HPO_4_ and 2 mM KH_2_PO_4_; pH 7.4) for 30 minutes and then treated with 0.05% (v/v) DMSO, 150 nM geldanamycin, 50 μM OY25 or OY29. The fluorescence was measured at λex = 485 nm and λem = 535 nm for 12 hours at 10-minute intervals using a SpectraMax M3 microplate spectrophotometer reader.

### ROS generation

To examine the effects of the ROS scavenger N-acetyl cysteine (NAC) on the cytotoxicity of the hit compounds, an MTT assay was carried out as described above, with minor modification. HCC70 cells were seeded at 5 × 10^3^ cells/well in a 96-well plate overnight and pre-treated with 30 mM NAC 30 minutes prior to 96-hour treatment with 0.01% (v/v) DMSO vehicle control, 0.2 μM elesclomol positive control, OY25 or OY29 at their IC_50_ values.

### Griess assay for nitrite detection

HCC70 cells were seeded at 5 × 10^3^ cells/well in a 96-well plate overnight and treated for 24 hours with 0.1% (v/v) DMSO vehicle control, 10 µg/mL phorbol-12-myristate-13-acetate (PMA), OY25 or OY29 (10, 50 and 100 µM). Media was harvested and incubated with the Griess reagents as previously described [44]. A standard curve was prepared from sodium nitrite and absorbance measured at 540 nm using a Synergy 2 Mx microtitre plate reader (Biotek Instruments Inc., USA) and nitrite concentration calculated from the standard curve linear regression generated in GraphPad Prism.

#### UV-Vis DNA titration assay.

The ability of the ferrocene-containing thiazolidine-2,4-dione compounds to bind to DNA was assessed as previously described [40,43]. Briefly, 100 ng/µL of Calf Thymus DNA (CT-DNA) was incubated with either 0.2% (v/v) DMSO vehicle control or OY25 and OY29 compounds (10, 50 and 200 µM) in a 100 µL reaction volume in a 96-well UV-Vis plate for 10 minutes. The absorbance was measured at wavelengths ranging from 220–350 nm. The binding constant (Kb) was calculated to quantify the affinity of the compounds for CT-DNA using a linear reciprocal plot based on the following equation (1) [45–47]:


$$\frac{1}{A{-A}_{0}}=\frac{1}{A_{\infty }-A_{0}}+\frac{1}{Kb[A_{\infty }-A_{0}]}\times \frac{1}{\left[C\right]}\mathrm{\backslash ]}$$ (1)

where A_0_ is the absorbance of DNA in the absence of the compounds, A is the absorbance of the compounds containing fixed amounts of DNA at different concentrations at 260 nm, and A ∞ is the theoretical final absorbance of the DNA-compound complex. From the linear plot 1/A-A_0_ vs 1/[C], the Kb was calculated as the ratio of the intercept to the slope.

### Agarose gel electrophoresis assessment of DNA binding

The ability of hit compounds to interact with human genomic DNA (gDNA) isolated from HCC70 cells was assessed as previously described [48]. Briefly, compounds OY25 and OY29 (10, 50 and 200 µM), 0.2% (v/v) DMSO vehicle control or 200 µM cisplatin positive control were incubated with 100 ng of gDNA at 37 °Ϲ for 4 hours, followed by agarose gel (0.8% [w/v], containing 0.5 μg/mL ethidium bromide) electrophoresis in 1 × TAE buffer. The DNA was visualized, and average fluorescence intensity determined as previously described [48].

### Competitive DNA binding assays

The ability of the hit compounds to compete with Hoechst 33342 or methylene blue for binding to CT-DNA was assessed as previously described [40,43]. OY25 and OY29 compounds (10, 50 and 200 µM) or 0.2% (v/v) DMSO vehicle control were incubated with a final concentration of 1 µg/mL of Hoechst 33342 or 15 µg/mL of methylene blue in the presence or absence of 100 ng/µL CT-DNA in a black-walled clear bottom 96-well plate for 10 minutes. Cisplatin (200 µM) was used as a positive control in the competitive DNA intercalation study with methylene blue dye.

### DNA docking studies

Docking studies were performed using Autodock Tools v1.5.6 [49] and AutoDock Vina [50,51] (Freeware maintained by Centre for Computational Structural Biology, Scripps Institute, USA) using a dodecamer B-DNA structure (5′-D(*CP*GP*CP*GP*AP*AP*TP*TP*CP*GP*CP*G)-3′) from RCSB Protein Data Bank (PDB: 129D) that has been complexed with a minor groove binder Hoechst 33342. The DNA structure was prepared for docking by removing water molecules and Hoechst 33342, followed by processing the DNA structure by adding all hydrogen atoms and merging non-polar hydrogen using Autodock Tools. Gasteiger charges and Autodock4 atom type (AD4 type) were calculated and assigned, respectively. The torsions of the compound (ligand) were then fixed, and a grid box was set to cover the DNA structure by adjusting the xyz co-ordinates and dimensions. Thereafter, docking was carried out using Autodock Vina, where the DNA and compounds were subjected to a configuration file followed by running a code in cmd terminal. Results of the lowest binding energy (kcal/mol) models were visualized using PyMOL (The PyMOL Molecular Graphics System, Schrodinger LLC, New York) and Discovery Studio Visualizer, where the receptor and ligand interactions were determined. Schematic diagrams of the receptor-ligand interactions were then visualized using Ligplot+ v1.4.5 (European Bioinformatics Institute, UK) [52].

### Indirect immunofluorescence staining using confocal microscopy

HCC70 cells were seeded into 18 well µ-Slide (Ibidi) chambers overnight and treated with 0.05% (v/v) DMSO vehicle control, 20 µM H2O2, 50 µM OY25 or OY29 for 2 hours. The immunofluorescence staining was carried out as previously described [53] with modification, where cells were fixed in ice-cold methanol and permeabilized using 0.1% (v/v) Triton^TM^ X-100. The corrected total cell fluorescence (CTCF) was used to calculate the intensity of the total cellular fluorescence in the nucleus using the equation: Integrated density − area of cell × mean fluorescence of background. The number of particles in the nucleus was also calculated according to the fluorescence intensity seen in the nucleus (CNCF).

### Development of a HeLa stable cell line expressing Apple-53 BP1 trunc

A polyclonal stable cell line was developed by transfecting HeLa cells (seeded at 2 × 10^5^ cells/well in a 6-well plate) with 2 μg of Apple-53 BP1 trunc plasmid with X-tremeGENE^TM^ HP DNA transfection reagent (1:2) in OptiMEM^TM^ reduced serum medium for 48 hours. Positive clones were selected using 2 μg/mL puromycin. For the analysis of the hit compounds on DNA damage and repair, the stable cells were seeded on coverslips at 5 × 10^4^ cells/well in a 24-well plate overnight and treated with either the DMSO vehicle control, oxaliplatin, OY25 or OY29 for 2 hours. Cells were then fixed with ice-cold methanol and visualized under the Olympus BX60 fluorescence microscope, and images taken with an Olympus DP72 digital camera using cellSens Entry software. The number of particles and cells with particles were quantified using ImageJ as described above.

### Combination assay for the analysis of synergistic relationships between compounds

Synergistic relationships between OY25 or OY29 compounds with known inhibitors such as camptothecin, etoposide and AZD2461 were investigated in HCC70 cells using the resazurin assay described above. Cells were treated in combination of the compounds and relevant inhibitor either, in a constant and non-constant ratio [54] for 96 hours. The ratios were calculated relative to the highest concentration of single treatments of either the compounds or inhibitors at which the fluorescence readings reached a plateau (100% death). The data was analyzed using CompuSyn software v.1 as previously described [55] where the dead cells and fraction affected were calculated as: Dead cells = average fluorescence of DMSO or untreated cells − average fluorescence treated cells, the dead cells were then used to calculate the effect or fraction affected (Fa), where Fa = Dead cells/DMSO or untreated cells. The Fa was inserted into CompuSyn software, where combination indexes (CI) were developed from constant ratio of combined drugs and algorithms.

### *In silico* analysis of ADME parameters

*In silico* ADME properties, which encompass physicochemical properties and drug-likeness parameters of the compounds OY24, OY25, and OY29 were calculated using the open-access SwissADME web tool (www.swissadme.ch, Swiss Institute of Bioinformatics®, Lausanne, Switzerland) [56] as previously described [57].

## Results and discussion

### *In vitro* cytotoxic analysis

Previously, we reported the synthesis and biological activity of OY24−29 (S1 Table) against *Plasmodium falciparum* and *Trypanosoma brucei brucei*, causative agents of malaria and human African trypanosomiasis, respectively [37]. Rescreening of ferrocenyl-containing thiazolidine-2,4-dione derivatives for cytotoxicity against the HCC70 triple-negative breast cancer cell line revealed three compounds displaying IC_50_ values below 10 μM (S1 Table). In particular, OY29 exhibited the lowest IC_50_ value of 5.59 ± 1.24 μM, followed by OY25 and OY24 with IC_50_ values of 7.54 ± 1.07 μM and 7.86 ± 1.37 μM (Table 1), respectively. The rest of the compounds showed moderate activity with IC_50_ values in the range of 20–45 μM (S1 Table). These compounds are structurally similar with the variation only at the carboxamide region of the compound. Replacement of the piperidine moiety present in OY29 with pyrrolidine (OY26), morpholine (OY27) and thiomorpholine (OY28) reduced the toxicity against the HCC70 cell line. Considering their low micromolar toxicity in TNBC cells, compounds OY24, OY25 and OY29 were also evaluated *in vitro* against the non-tumourigenic breast epithelial cell line (MCF-12A) to assess selective toxicity against cancer versus non-cancer cells. Compounds OY25 and OY29 displayed a degree of selectivity for HCC70 cells (SI values of 2.22 and 4.44), while OY24 was non-selective, resulting in its exclusion from further analysis. Thus, compounds OY25 and OY29 were further assessed in another TNBC cell line (HCC1937), a Luminal A (non-TNBC) breast cancer cell line (MCF-7), a human cervical cancer cell line (HeLa) and a mouse embryonic fibroblast cell line (MEF-1). The MEF-1 cell line is routinely used in biological screening assays as a model of normal (non-cancerous/non-tumourigenic) cells. OY25 exhibited IC_50_ values of lower than 10 μM in the four cancer cell lines, while being non-toxic to MEF-1 cells. Contrary, OY29 showed reduced activity against all cell lines (except HCC70) in comparison to OY25. Similarly, compound OY29 was non-toxic against MEF-1 cells. Geldanamycin (GA) and paclitaxel (PTX), established chemotherapeutic agents employed in this study as reference drugs, displayed IC_50_ values in the nanomolar range against all cell lines. Although GA displayed poor selectivity for cancer cells, overall, the selectivity of PTX was comparable to that of thiazolidine-2,4-dione derivatives OY25 and OY29 (Table 1). Previously, Metwally et al., reported a series of two different thiazolidinedione scaffolds, where the thiazolidinedione ring was located either in the terminal or middle of the molecules and were tested for cytotoxicity activity on MDA-MB-231 TNBC cells with IC_50_ values ranging from 4.5–87.2 µM and 5.9–39.7 µM, respectively [58]. The thiazolidine-2,4-diones bearing sulfonylthiourea moieties were tested against human breast cancer cell line MCF-7, revealing IC_50_ values ranging from 7.78–62.4 µM [59]. Romagnoli et al., synthesized thiazolidine-2,4-dione derivatives with a series of N-3-substituted-5-arylidene bearing the α-bromoacryloylamido moiety and were screened for toxicity against Hela cells, and IC_50_ values from 0.80–8.9 µM were obtained [60].

**Table 1:** *In vitro* antitumour screening of ferrocenyl thiazolidine-2,4-dione derivatives and control compounds against cancerous and non-cancerous cell lines.

IC_50_ (µM) ± SD						
Compound	HCC70	HCC1937	MCF-7	HeLa	MCF-12A	MEF-1
OY24	7.86 ± 1.37 **SI: 0.3397**^[a]^	ND^[b]^	ND	ND	2.67 ± 1.21	ND
OY25	7.54 ± 1.07 **SI: 2.2188**	6.78 ± 1.05 **SI: 2.4697**	2.19 ± 1.16 **SI: 7.6206**	5.60 ± 1.18 **SI: 2.9911**	16.8 ± 1.37	NT > 500^[c]^
OY29	5.59 ± 1.24 **SI: 4.4359**	45.7 ± 1.12 **SI: 0.5426**	28.8 ± 1.41 **SI: 0.8629**	24.2 ± 1.15 **SI: 1.0239**	24.8 ± 1.17	NT > 500
GA (nM)	33.5 ± 1.14 **SI: 0.3137**	378.9 ± 1.12 **SI: 0.0277**	60.6 ± 1.08 **SI: 0.1734**	ND	10.5 ± 1.07	42.4 ± 1.14 **SI: 0.2478**
PTX (nM)	3.91 ± 1.03 **SI: 4.124**	ND	2.40 ± 1.10 **SI: 6.7138**	2.69 ± 1.12 **SI: 5.9918**	16.2 ± 1.08	ND

[a] SI: selectivity index. [b] ND: not determined. [c] NT: non-toxic.

### Estimation of DNA damage generation in HCC70 cells

**Fig 1:**
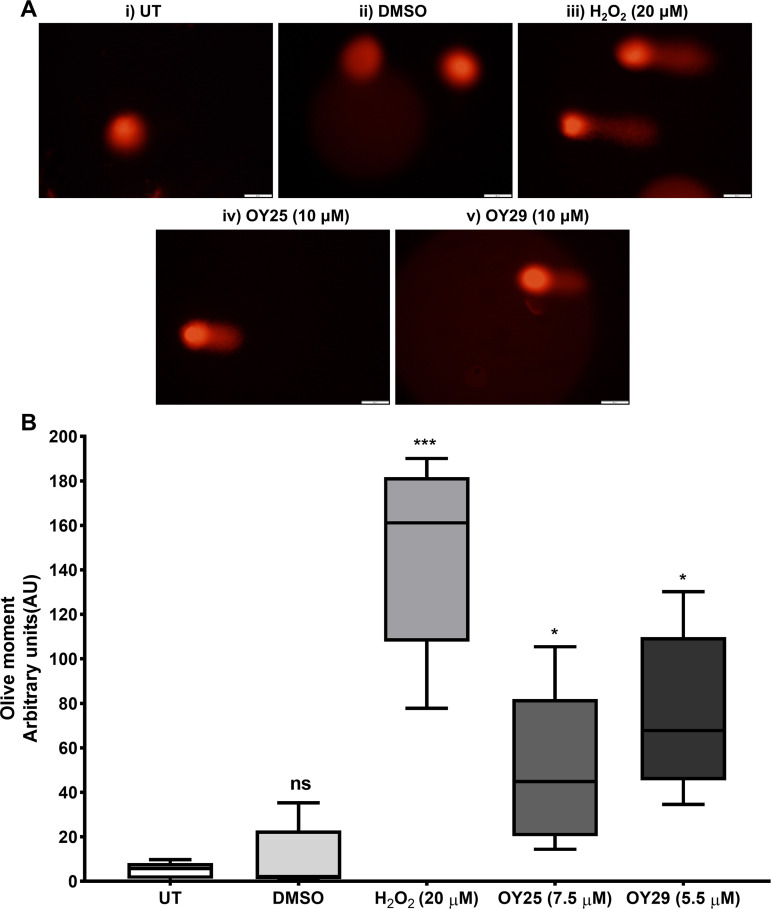
Alkaline comet assay indicating ferrocene thiazolidine-2,4-dione derivatives OY25 and OY29 induce DNA damage in HCC70 cells. (A) Images of individual propidium iodide-stained nuclei were obtained using an Olympus fluorescence microscope (Japan) under a wide green filter tube, λ_ex_ = 510 nm and λ_em _= 590 nm for the red fluorophore, at 20 × magnification. The images were analyzed using an Image J plugin, OpenComet. (B) The box-and whisker plot of the Olive moment generated using GraphPad Inc (USA). Statistical analysis was carried out relative to the untreated (UT) using One-way ANOVA with Bonferroni’s multiple comparisons test, where ns = not significant, *p < 0.05 and ***p < 0.001. Representative images and data shown (n ≥ 20). Scale bars represent 50 μm.

To investigate the potential mode of action of ferrocene-containing thiazolidine-2,4-dione derivatives, we initially studied their ability to induce DNA damage using the alkaline comet assay, which detects DNA single or double-strand breaks and alkali-labile lesions [40,61]. Treatment of HCC70 cells with OY25 (Fig 1Aiv) and OY29 (Fig 1Av) resulted in distinct comet tails in both cases, and a significant increase in Olive moment (Fig 1B) compared to the vehicle-treated control was observed. Consistent with the H_2_O_2_ positive control, the data suggested that OY25 and OY29 induce significant levels of DNA damage in TNBC cells.

Previous studies have reported that the ferrocene moiety may be activated by endogenous H_2_O_2_ (Fenton-type reaction) in cancer cells leading to production of hydroxyl (٠OH) ions resulting in DNA damage [62–64]. Intracellularly, ferrocene unit undergo one electron oxidation to produce ferrocenium radical cation resulting in production of reactive oxygen species (ROS) under physiological conditions [63,65]. In another study by Rodrigues and colleagues, 5-(2-Bromo-5-methoxybenzylidene)-thiazolidine-2,4-dione showed significant DNA damage induction in the NCI-H292 lung cancer cell line [66].

**Fig 2:**
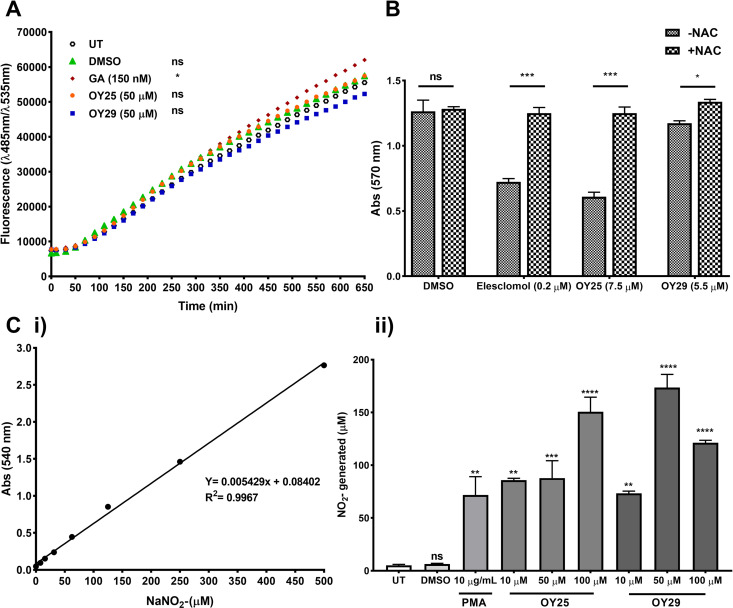
Assessment of ROS induction by OY25 and OY29 in HCC70 cells. (A) DCFDA assay in HCC70 cells. GA: Geldanamycin, positive control. (B) N-acetyl cysteine (NAC) free radical scavenger MTT assay. (C) Griess assay showing detection of nitrites (NO_2_-) in HCC70 cells treated with OY25 or OY29. (i) Standard curve graph using sodium nitrite (NaNO_2_-), (ii) nitrites (NO_2_-) generation in treated HCC70 cells. Statistical analysis was carried out relative to the untreated cells (UT) by One-way ANOVA with Dunnett’s multiple comparisons test, where ns = not significant, *p < 0.05, **p < 0.01, ***p < 0.001 and ****p < 0.0001. Data shown are averages (n = 3, with SD) and are representative of biological duplicate experiments.

To further characterize the observed cytotoxicity and DNA damage effects of OY25 and OY29 in TNBC cells, we assessed the effect of achieved thiazolidine-2,4-dione derivatives on inducing ROS [63]. In a 2,7-dichlorofluorescin diacetate (DCFDA) assay (Fig 2A), OY25 (orange circles) and OY29 (blue squares) had no significant effect on ROS levels compared to the untreated (open black circles) or dimethyl sulphoxide (DMSO) vehicle control (green triangles). The positive control GA [67] significantly induced ROS levels from approximately 400 minutes (shown in maroon). On the other hand, like the elesclomol positive control [68] the toxicity of the thiazolidine-2,4-dione derivatives in HCC70 cells was reduced in the presence of a ROS scavenger N-acetyl cysteine (NAC, Fig 2B), which interacts with ROS such as٠OH and H_2_O_2_, leading to cell survival [69,70]. Collectively, these results confirm that the toxicity of OY25 and OY29 is mediated, at least in part, by the generation of ROS. In a previous study, ROS scavengers NAC and ascorbic acid were found to have a protective effect on the production of ROS of thiazolidine-2,4-dione derivatives, ∆2-pioglitazone in colon cancer cells, HCT116 and HT29 [71]. In addition, to detect sufficient nitrite (NO_2_-) generation in the Griess assay [72], the concentration of the compounds was increased up to 100 µM. The results showed significant detectable levels of NO_2_- in cells treated with OY25 and OY29 (Fig 2C) similar to that of the protein kinase C activator (PMA) positive control [73]. Although a non-dose dependent increase was observed in cells treated with OY29, this effect could be due to the high concentration of the compound (100 µM), resulting in cytotoxic effects in cancer cells, leading to cell death [74,75]. NO_2_- reacts with other free radicals, which can lead to genotoxic effects [76]. These findings suggest that the cytotoxicity and the DNA damage induced by OY25 and OY29 may be linked to the ferrocene group generating radicals resulting in the production of ROS and RNS, despite the failure to detect increased ROS via the DCFDA assay. The failure to detect an increase in ROS of the ferrocene-containing thiazolidine-2,4-dione derivatives via DCFDA assay could be due to a few limitations of the ROS assay, and a common limitation is the use of organic solvents, such as DMSO and ethanol, to dissolve test compounds. The test compounds OY25 and OY29 were dissolved in DMSO, and according to Acker et al., these organic solvents are strong hydroxyl scavengers, which interfere with the assay by reducing the amount of ROS produced, potentially leading to an underestimation of ROS generation [77,78].

### Assessment of DNA interaction in HCC70 cells

**Fig 3:**
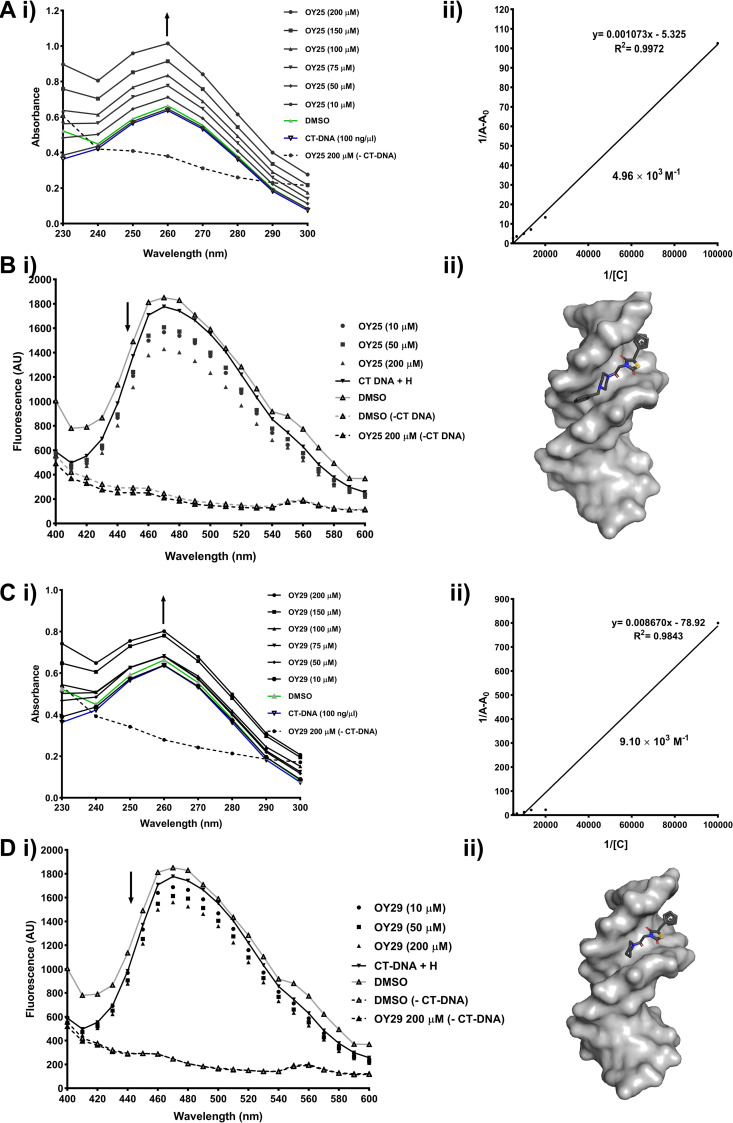
OY25 and OY29 interact directly with bovine DNA as minor groove binders. (A and C) UV–Vis absorption spectra (i) of calf thymus (CT) DNA (100 ng/μL) incubated with (A) OY25 and (C) OY29 (10–200 μM) measured at wavelengths ranging from 230–300 nm and (ii) linear plot of 1/[A-A_0_] vs 1[C], the reciprocal of the absorbance of the CT-DNA-compound complex and the concentrations of the compounds. Bi,Di) Fluorescence emission spectra (400–600 nm) of 100 ng CT-DNA in the presence of (B) OY25 and (D) OY29 at concentrations of 10, 50 and 200 µM measuring competition with 1 µg/mL Hoechst for minor groove binding ability. Data shown as averages (n = 3, technical replicates), and representative of three biological replicates. OY25 (Bii) and OY29 (Dii) (dark grey with coloured atoms) docked in the minor groove site of a surface representation of DNA (shown in light grey).

We conducted DNA binding studies to determine if there is any correlation between induction of DNA damage by OY25 and OY29 and direct binding of the same compounds to DNA. The UV-Vis absorbance spectra (Fig 3A and C) and fluorescence of ethidium bromide-stained DNA (S1A Fig) in the presence and absence of the test compounds were compared. When OY25 and OY29 were incubated with CT-DNA, a dose-dependent increase in the absorbance values between 240–280 nm were observed above that of the CT-DNA alone and DMSO vehicle control, with 37.25% and 20.54% hyperchromic effect at 200 µM (Fig 3A and C), respectively. The binding of compounds to DNA causes a hyperchromic shift at 260 nm as nucleotides become exposed [79,80] and is a characteristic of conformational alteration of the DNA double helix [79,81]. Therefore, the UV-Vis absorption suggests that OY25 and OY29 change the DNA conformation by directly binding to the DNA, with Kb values of 4.96 × 10^3^ M^‐1^ and 9.10 × 10^3^ M^‐1^, respectively. Previous reports indicate that ß-carboline-linked 2,4-thiazolidinedione derivative displays CT-DNA binding ability, causing hyperchromic shifts with binding affinity of 1.40 × 10^5^ M^‐1^ [82]. The literature suggests that this derivative has a greater affinity for DNA, as compared to OY25 and OY29. A binding constant in the order of magnitude of 1 × 10^4^ is considered to represent weak to moderate binding [83,84], thus it can be concluded that the binding of OY25 and OY29 to CT-DNA falls in this category of weak to moderate binding to/affinity for the DNA.

In the ethidium bromide competition agarose gel electrophoresis assay, both OY25 and OY29 were able to displace ethidium bromide to interact directly with human gDNA, as reflected by significantly reduced fluorescence intensity of ethidium bromide staining compared to that of the DMSO control, with OY29 resulted in a greater reduction in the intensity of ethidium bromide staining as compared to OY25 (S1A Fig).

Following confirmation of direct binding of the compounds to DNA, the mode of binding was assessed by competitive binding studies with DNA intercalator methylene blue (MB) [85], and DNA minor groove binder Hoechst 33342 [86]. The MB DNA competition studies for OY25 and OY29 revealed that incubation of compounds with the DNA at the highest concentration of 200 µM yielded an increase in fluorescence compared to that of the DNA-MB complex alone (S1B and S1E Fig), suggesting that these compounds could be acting as intercalators as observed for the cisplatin, an agent that intercalates into the DNA covalently [87]. However, DNA-MB-DMSO vehicle control complex at 200 µM was shown to have a high fluorescence compared to that of the DNA-MB and DNA-MB-compounds complexes. A study by Shahinyan and colleagues, demonstrated that MB is sensitive to DMSO [88]. MB dimerizes in water and the addition of DMSO prevents the dimerization of MB resulting in high fluorescence intensity, because of the increase of free monomeric dye molecules [88]. Since OY25 and OY29 were dissolved in DMSO, it is possible that these results could thus suggest that the increased fluorescence observed in the MB-compound complex could be due to the sensitivity of MB to DMSO, thus negating intercalation of the compounds with the DNA.

In the Hoechst 33342 competition assay, the fluorescence intensity of the DNA-Hoechst complex was decreased in the presence of both OY25 and OY29 at all concentrations 10, 50 and 200 µM (Fig 3Bi and Di) compared to the DNA-Hoechst 33342 complex (shown as a solid black line) and DMSO vehicle control (shown as a grey line), indicating that OY25 and OY29 are able to displace Hoechst 33342 from CT-DNA and suggesting that the compounds are minor groove binders. The fluorescence quenching of OY25, 31.72% was higher compared to that of OY29, 20.85% at 200 µM calculated using F_0_–F/F equation where F_0_ and F are fluorescence readings of Hoechst 33342-DMSO vehicle control complex and Hoechst-compound complex, respectively. The mode of binding of OY25 and OY29 was confirmed by molecular docking of compounds to a B-DNA, a right-handed helical structure dodecamer structure (PDB:129D) [89,90] which was complexed with Hoechst 33342. The compound-DNA interaction of the OY25-DNA (Fig 3Bii) and OY29-DNA-complexes (Fig 3Dii) were generated using Discovery Studio Visualizer, Ligplot+ and PyMOL. Both OY25 and OY29 appear to interact within the minor groove site of the DNA (Fig 3Bii and 3Dii). S1C, S1D, S1F and S1G Fig show the residues of the DNA involved in interactions with OY25 and OY29, respectively. The results of the docking studies indicated that OY25 exhibited moderate binding affinity for the DNA of −8.4 kcal/mol [91]. A potential hydrogen bond formed between the oxygen atom of the compound and guanine 10 of the DNA at a moderate bond distance of 3.07 Å (S1C and D Fig), a distance of 2.5–3.2 Å is considered the limit of moderate hydrogen bond length [92,93]. OY29 exhibited a moderate binding affinity for the DNA of −8.1 kcal/mol, with a potential hydrogen bond formed between oxygen of the compound and guanine 4 with a moderate bond distance of 3.10 Å (S1F and G Fig). When Hoechst 33342 was complexed with the crystallized DNA dodecamer structure, it was found to lie at the central AATT rich site and interacted with Guanine 4-Cytosine 21 (G4-C21) and Guanine 16-Cytosine 9 (G16-C9) base pairs [94–96]. Since OY25 was shown in the docking studies to potentially interact with Cytosine 9 and 16 and OY29 to interact with Cytosine 21 (S1 Fig and S2 Table), this also suggests that the OY25 and OY29 are indeed binding in the minor groove and interacting with some of the same base pairs that Hoechst 33342 interacts with.

**Fig 4:**
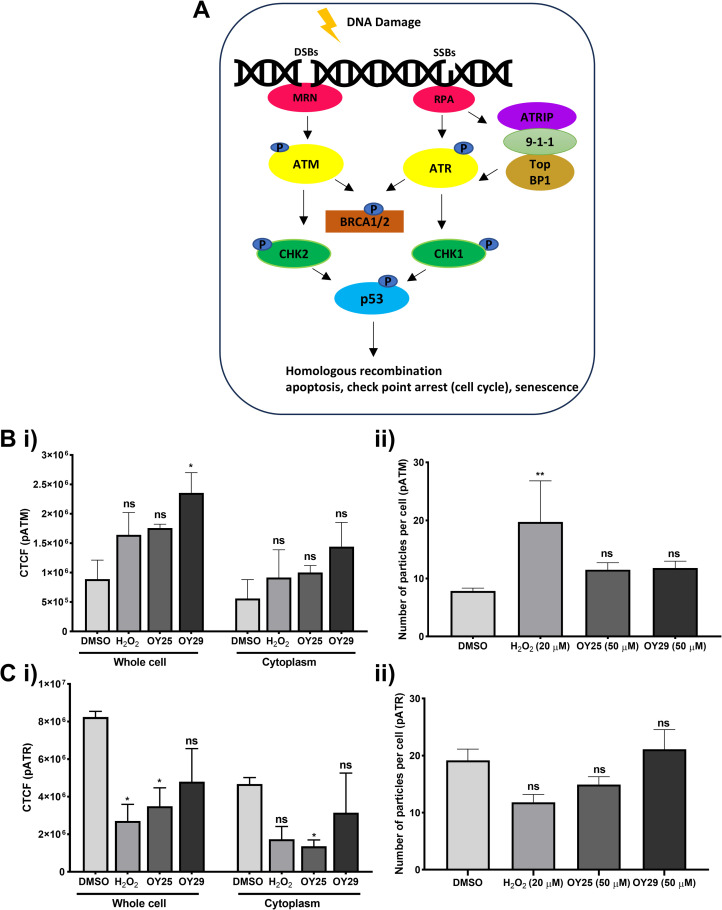
Interference of OY25 and OY29 with DNA damage response proteins in HCC70 cells. (A) Schematic diagram of the DNA damage response pathway. The response to double-strand breaks (DSBs) and single-strand breaks (SSBs) involves the binding of MRN and RPA, which activates and phosphorylates ATM and ATR, ultimately, leads to downstream activation of other proteins such as, BRCA1/2, CHK1, CHK2 and p53. DNA DSBs and SSBs are then repaired by means of cell cycle arrest and the homologous recombination pathway. Image adapted from [100] and [101]. (Bi, Ci) Average corrected total cellular fluorescence intensity (CTCF) and the Bii, Cii) number of particles (punctae) per cell were determined for pATM/pATR using Image J Software, data shown are averages (n ≥ 30, with SEM). Statistical analyses were carried out relative to the DMSO vehicle control by One-way ANOVA with Bonferroni’s multiple comparisons test, where ns = not significant and *p < 0.05.

The strategy of targeting the DNA repair pathway has been shown to be a powerful tool to treat cancer [97]. In an attempt to characterize the robust DNA damage response induced by the selected compounds, the effects of these compounds on key DNA damage-induced repair proteins were assessed (S2 Fig). Ataxia telangiectasia mutated (ATM) and ataxia telangiectasia mutated and Rad3-related (ATR) protein kinase are recruited, stimulated, and phosphorylated upon induction of DNA double-strand breaks (DSBs) and single-strand breaks (SSBs), respectively. Both ATM and ATR phosphorylate downstream proteins, including BRCA1/2 (breast cancer 1/2 gene), CHK1 (checkpoint kinase 1), CHK2 and p53 (tumour protein 53) (Fig 4A) [98]. Immunofluorescence analysis for pATM and pATR (shown in green, S2A and B Fig) revealed the elevation of pATM levels upon treatment with H_2_O_2_, OY25 and OY29 compared to the vehicle control, although this increase was statistically significant only in the case of OY29 treatment in the whole cell (Fig 4Bi and Ci), while the number of ATM puncta (Fig 4Bii and Cii), representing areas of active DNA repair, was not significantly altered upon treatment. On the other hand, a significant decrease in pATR levels, but not punctae, was observed in cells treated with H_2_O_2_ and OY25. The increase in cytoplasmic pATM levels in response to OY25 and OY29 could be activated by ROS, as ATM activation in the cytoplasm by ROS has previously been reported [99]. The alterations in the levels of these phosphoproteins could be a response to the DNA damage induced by the compounds or could represent interference with the DNA repair pathway, the latter of which would result in a dual mode of action of these compounds if they were both able to induce DNA damage and interfere with DNA repair. The increase and decrease of these phosphoproteins upon treatment with these compounds suggests the involvement of both the ATM and ATR pathways triggered by both DSBs and SSBs.

**Fig 5:**
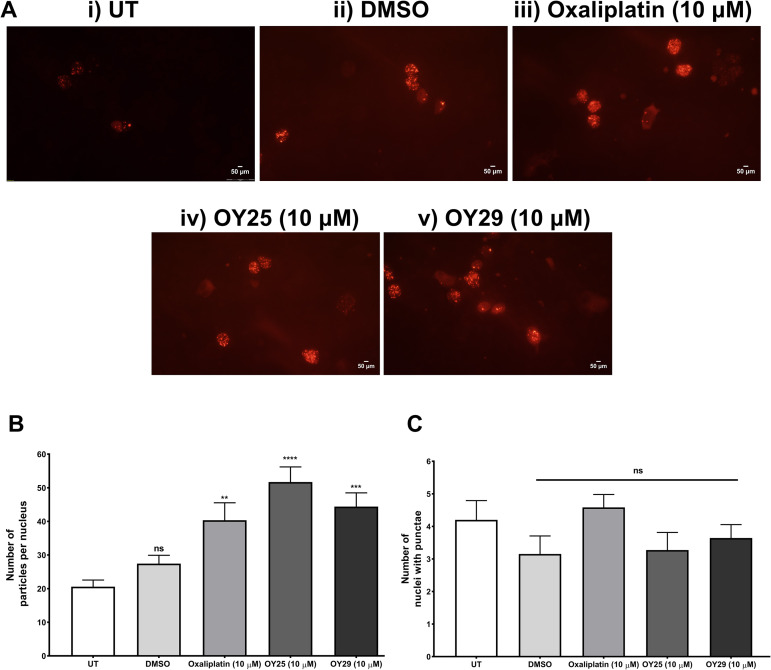
OY25 and OY29 induce DNA double-strand breaks and repair as assessed using a p53 binding protein reporter system. (A) Fluorescence images obtained using an Olympus fluorescence microscope under a wide green filter tube, λ_Ex _= 510 nm and λ_Em =_ 590 nm for mApple signal, red fluorescence represents 53 BP1 staining. The images were analyzed using Image J to quantify (B) the number of particles per nucleus and (C) the average of nuclei with punctae. Statistical analyses were carried out relative to the untreated (UT) by One-way ANOVA with Dunnett’s multiple comparisons test, where ns = not significant, *p < 0.05, ***p < 0.001 and ****p < 0.0001. Data shown are averages (n ≥ 30 nuclei, with SEM).

A polyclonal stable cell line expressing p53 binding protein-1 (Apple-53 BP1trunc) was generated in HeLa cells to measure DNA damage by DSBs accumulation [102]. Like oxaliplatin (used as a positive control), OY25 and OY29 treatment cause a significant increase in the number of 53 BP1 puncta/particles per cell/nucleus (Fig 5Aiii–v and B) compared to the untreated and the DMSO vehicle controls. The number of cells with 53 BP1 puncta was unaltered across all the treated cells (Fig 5C), compared to the UT, indicating that, while most cells display such puncta and DNA damage repair in progress, as expected, it is the extent of such sites that varies upon induction of DNA damage by a compound. Since p53BP is a transcriptional coactivator with p53 and is involved in DSB repair, the induction of p53BP puncta upon treatment with OY25 and OY29 could again suggest their involvement in modulating DNA damage repair pathways.

**Fig 6:**
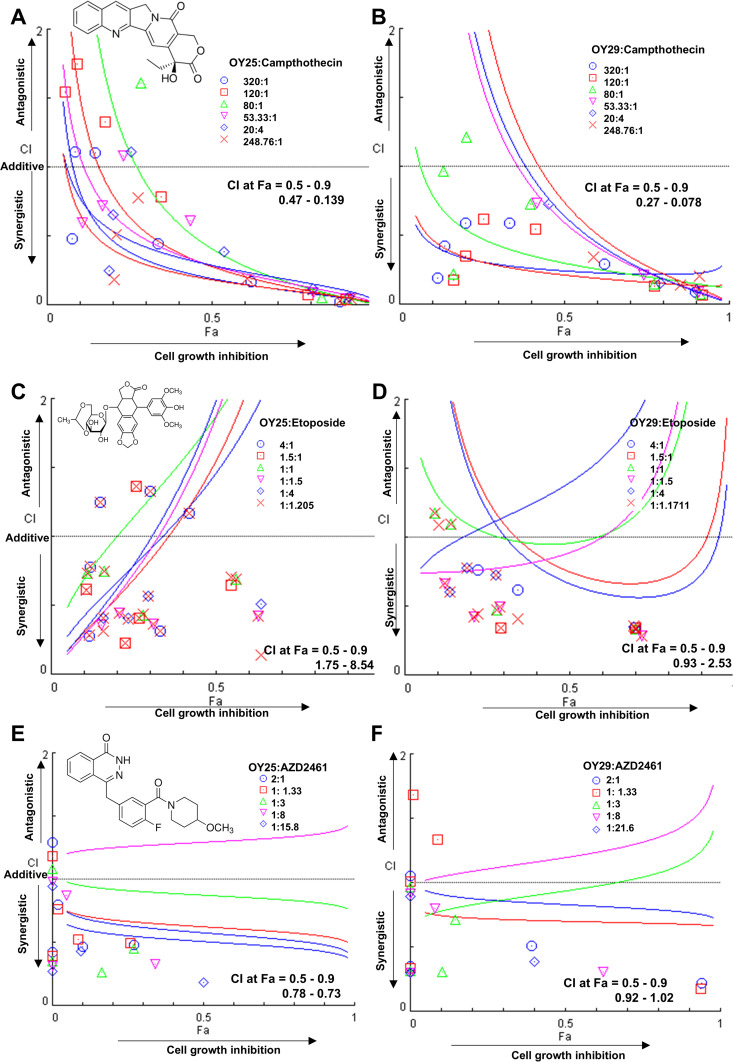
Combination studies of OY25 and OY29 with known DNA damage-inducing drugs. Structures of the DNA damage-inducing agents and combination index (CI) vs fraction of cells affected (Fa) plot for: (A) OY25 and camptothecin, (B) OY29 and camptothecin, (C) OY25 and etoposide, (D) OY29 and etoposide, (E) OY25 and AZD2461 and (F) OY29 and AZD2461. CI vs Fa Plots were generated using CompuSyn software [55]. The effect of the drug combination is defined as follows synergistic CI < 1, additive CI = 1 or antagonistic CI > 1. Data shown are averages (n = 3, technical triplicates).

Finally, the effect of OY25 and OY29 on DNA damage and repair was assessed indirectly by means of synergy studies using known inhibitors of these cellular processes. Drug combination is used in treating diseases such as cancer, with the aim of achieving synergistic effects and doses with reduced toxicity or a delay of drug resistance [103,104]. HCC70 cells were treated with the compounds in combination with camptothecin or etoposide, as topoisomerase I and II inhibitors, respectively. Topoisomerase inhibitors block the re-ligation of the transient breaks made by the topoisomerase enzyme during replication and transcription, resulting in DNA damage [105]. Camptothecin induces both SSBs and DSBs [106] and etoposide induces DSBs [107]. The Combination Index (CI) vs Fraction Affected (Fa) plots for OY25 and OY29 in combination with camptothecin show most of the combination treatment points and the trendlines below the CI = 1 cut-off, indicating a synergetic effect of the combination of the hit compounds with the topoisomerase I inhibitor (Fig 6A and B). On the other hand, the CI vs Fa plot for OY25 and OY29 in combination with etoposide, show a spread of the combination treatment points above and below the line of CI = 1 cut-off, with CI at Fa values between 0.5–0.9 for the OY25:etoposide combination range from 1.75–8.54 and for the OY29:etoposide combination range from 0.93–2.53 (Fig 6C and D) indicating strong antagonism between OY25 and etoposide and a moderately antagonistic relationship between OY29 and etoposide.

To further explore the potential consequences of DNA damage induction by the ferrocenyl thiazolidinedione compounds and their therapeutic potential, synergy cytotoxicity studies were carried out with the Poly (ADP-ribose) polymerase-1 (PARP-1) inhibitor, AZD2461. PARP-1 inhibition using AZD2461 is a novel treatment approach for TNBC via the mechanism of synthetic lethality that exploits the weakened DNA repair machinery of these cells because of the high prevalence of mutations in the *BRCA1/2* and *TP53* genes [108–111]. To assess whether the combination of the hit compounds with a PARP-1 inhibitor would potentiate their toxicity, the cells were treated with OY25 and OY29 in combination with AZD2461, revealing a mildly synergistic effect for the combination of OY25 with PARP-1 inhibitor and an additive effect for OY29 when combined with the PARP-1 inhibitor.

In the presence of a PARP inhibitor, PARP is trapped on to the unpaired SSBs [110,112–114], resulting in DSBs due to the collapse of the replication fork. The DSBs are repaired by the HR repair pathway involving *BRCA1* and *BRCA2* genes [115,116]. Previous reports suggest that synergy may be observed where two pathways with a similar function or contributing to the same cellular process, in this case DNA repair, are being inhibited by the two compounds. In this case for OY25, it would point to involvement of the thiazolidine-2,4-dione derivatives in inhibiting DNA repair, and it is possible that this is via a different repair pathway to that in which PARP is involved. Compensatory activity between different DNA repair pathways is the basis of synthetic lethality and explains why synergy would be observed between compounds targeting different, potentially compensatory DNA repair pathways. Future work could involve assessing these effects in *TP53* and/or *BRCA1/2* wildtype TNBC cells to compare the effects on downstream proteins and the cross talk between pathways in such cell lines with those observed in the HCC70 cell line utilized in this study.

### Pharmacokinetic and pharmacodynamic profile

In clinical trial stages, the high attrition of drug molecules is attributed to undesirable pharmacokinetic properties including physicochemical parameters [56,117] The physiochemical properties and drug-likeness of selected compounds OY24, OY25 and OY29 were studied using *in silico* methods such as the web-based tool, SwissADME (http://www.swissadme.ch), [118] and the results are summarised as shown in S3 Table and S3 Fig.

Compounds OY24, OY25 and OY29 with promising *in vitro* antitumor activity (Table 1) exhibited acceptable drug-likeness properties. Despite the poor efficacy of most compounds (S1 Table), overall, the reported compounds showed parameters that are within the expected ranges described by Lipinski et al*.* [119] and only compound OY25 showed molecular mass > 500 g/mol threshold. The application of PAINS (Pan-assay interference compounds) filter showed the reduced likelihood (S3 Table) of compounds OY24, OY25 and OY29 acting as PAINS. The aqueous solubility of these compounds (S3 Table) was predicted to be moderately to highly soluble. All the compounds displayed acceptable topological polar surface area (TPSA) values below 140 Å^2^ accompanied by desirable gastrointestinal absorption (S3 Fig). The reported compounds showed no BBB penetration, which suggests that these compounds showed a reduced risk of potential CNS toxicity. Lastly, these compounds emerged as non-substrates of P-glycoprotein, which qualifies them as hits for further optimisation against TNBC.

## Conclusion

This study demonstrated that OY25 and OY29 have anticancer activity, being cytotoxic to TNBC cells *in vitro* in the low micromolar range. The cytotoxicity appeared to be dependent on ROS generation, and the compounds elicited a robust DNA damage response. Furthermore, OY25 and OY29 altered the level of key phosphoproteins in the homologous recombination repair pathway. The DNA-damaging effects of OY25 and OY29 are likely linked to their demonstrated ability to bind directly to DNA with a weak to moderate binding affinity, in the minor groove, with both *in silico* and *in vitro* analyses demonstrating that the compounds interact with some of the DNA base pairs that Hoechst 33342 interacts with and compete to displace Hoechst 33342, respectively. This leads to the hypothesis that OY25 and OY29 have a potential dual mechanism of action, both inducing DNA damage and interfering with DNA repair. The ADME predictions suggest that the compounds possess drug-like properties and show potential for further development with appropriate optimization. Overall, these ferrocene thiazolidine-2,4-dione derivatives have previously demonstrated antiplasmodial and antiparasitic activities, and in this study, exhibited anticancer activity, highlighting their potential as multi-target ligands against several human diseases.

## Supporting information

S1 Table Screening of six ferrocenyl thiazolidine-2,4-dione derivatives in HCC70 TNBC cell line. (DOCX)

S1 Fig Assessment of direct binding of OY25 and OY29 to DNA by competitive intercalation assays and molecular docking studies. (A) Ethidium bromide competition for assessment of interaction of OY25 and OY29 with human genomic DNA. (Ai) 0.8% (w/v) ethidium bromide-stained agarose gel containing DNA extracted from HCC70 breast cancer cells. + : cisplatin). (Aii) The average fluorescence intensity (n = 3, with SEM), representative of three biological replicates of the ethidium bromide staining of the DNA bands quantified using ImageJ and shown relative to the intensity of the DMSO control. Statistical analyis was carried out using One-way ANOVA with Bonferroni’s multiple comparisons tests, where ns = non-significant, **p < 0.01 and ***p < 0.001. (B and E) Fluorescence emission spectra (650–685 nm) of 100 ng calf thymus DNA (CT-DNA) in the presence of (B) OY25 and (E) OY29 at concentration of 10, 50 and 200 µM measuring competition with 15 µg/mL methylene blue for the ability to intercalate into DNA. Cisplatin was used as a positive control. Molecular docking of (C and D) OY25 and (F–G) OY29 to DNA (PDB: 129D). (C and F) DNA-ligand interaction of OY25 and OY29, respectively bound to DNA. (C) OY25 and (F) OY29 DNA binding site containing interacting residues in maroon, hydrogen bonds in green dashed lines and other non-bonding interaction/hydrophobic forces shown in either blue or purple dashed lines. Ligplot+ interaction map of docked (D) OY25 and (G) OY29 in the DNA binding site. (D and G) The ligands (OY25/OY29) are shown as ball and stick projections with the black balls representing carbon atoms, the purple line representing the bonds between connected atoms and the brown line representing the base interacting with the potential hydrogen bond formed. Red, blue, purple and yellow balls represent oxygen, nitrogen, phosphate and sulfur, respectively. Hydrogen bonds are shown as green dashed lines together with a bond length/distance in Angstrom (Å). The red bristle represents the non-ligand (bonding) interactions/residues (DNA) involved in hydrophobic interactions of the docked compounds (OY25/OY29) and the atoms of the DNA, corresponding to the hydrophobic forces exerted by the compounds on the DNA.
(TIF)

S2 Table Binding affinity and molecular interaction of OY25 and OY29 with DNA (PDB:129D). (DOCX)

S2 Fig Confocal microscopy assessment of the effects of OY25 and OY29 on pATR and pATM proteins in HCC70 cells. HCC70 cells were treated with 0.05% (v/v) DMSO vehicle control, 20 µM H_2_O_2_, 50 µM OY25 or OY29 for 2 hours. A-B Cells stained for (A) pATM and (B) pATR visualized under a Zeiss LSM780 Meta Confocal Microscope at λ_ex_ = 488 nm and λ_em_ = 562 nm laser and images taken (n ≥ 30, cells) using Zen Blue software (Zeiss). Green staining represents A) pATM and B) pATR with the nucleus shown in blue, following staining with Hoechst 33342.
(TIF)

S3 Table Predicted physicochemical properties and some drug-likeness parameters of ferrocenyl thiazolidine-2,4-diones OY24, OY25 and OY29. (DOCX)

S3 Fig BOILED-Egg diagram of compounds OY24, OY25 and OY29. Yellow illustrates BBB permeation, and white depicts gastrointestinal permeation. Non-substrate, P-glycoprotein (−) is represented by the red dot.
(TIF)

S1 Data Underlying data. (XLSX)
