# Supplementary material for: Ferrocene thiazolidine-2,4-dione derivatives cause DNA damage and interfere with DNA repair in triple-negative breast cancer cells
Source: PLoS One. 2025 Jul 17;20(7):e0328155. doi: 10.1371/journal.pone.0328155 (PMC12270111; doi:10.1371/journal.pone.0328155)
Supplement: S1 Table — (DOCX) [file pone.0328155.s001.docx]

**S1 Table. Screening of six ferrocenyl thiazolidine-2,4-dione derivatives in HCC70 TNBC cell line.**

| **Compound** | **Chemical Structure** | **IC_50_ ± SD (µM)**  **(HCC70 cells)** |
| --- | --- | --- |
| OY24 |  | 7.86 ± 1.37 |
| OY25 |  | 7.54 ± 1.07 |
| OY26 |  | 22.19 ± 22.18 |
| OY27 |  | 45.87 ± 1.31 |
| OY28 |  | 20.59 ± 1.29 |
| OY29 |  | 5.59 ± 1.24 |
