# Supplementary figures and images for: Ferrocene thiazolidine-2,4-dione derivatives cause DNA damage and interfere with DNA repair in triple-negative breast cancer cells
Source: PLoS One. 2025 Jul 17;20(7):e0328155. doi: 10.1371/journal.pone.0328155 (PMC12270111; doi:10.1371/journal.pone.0328155)

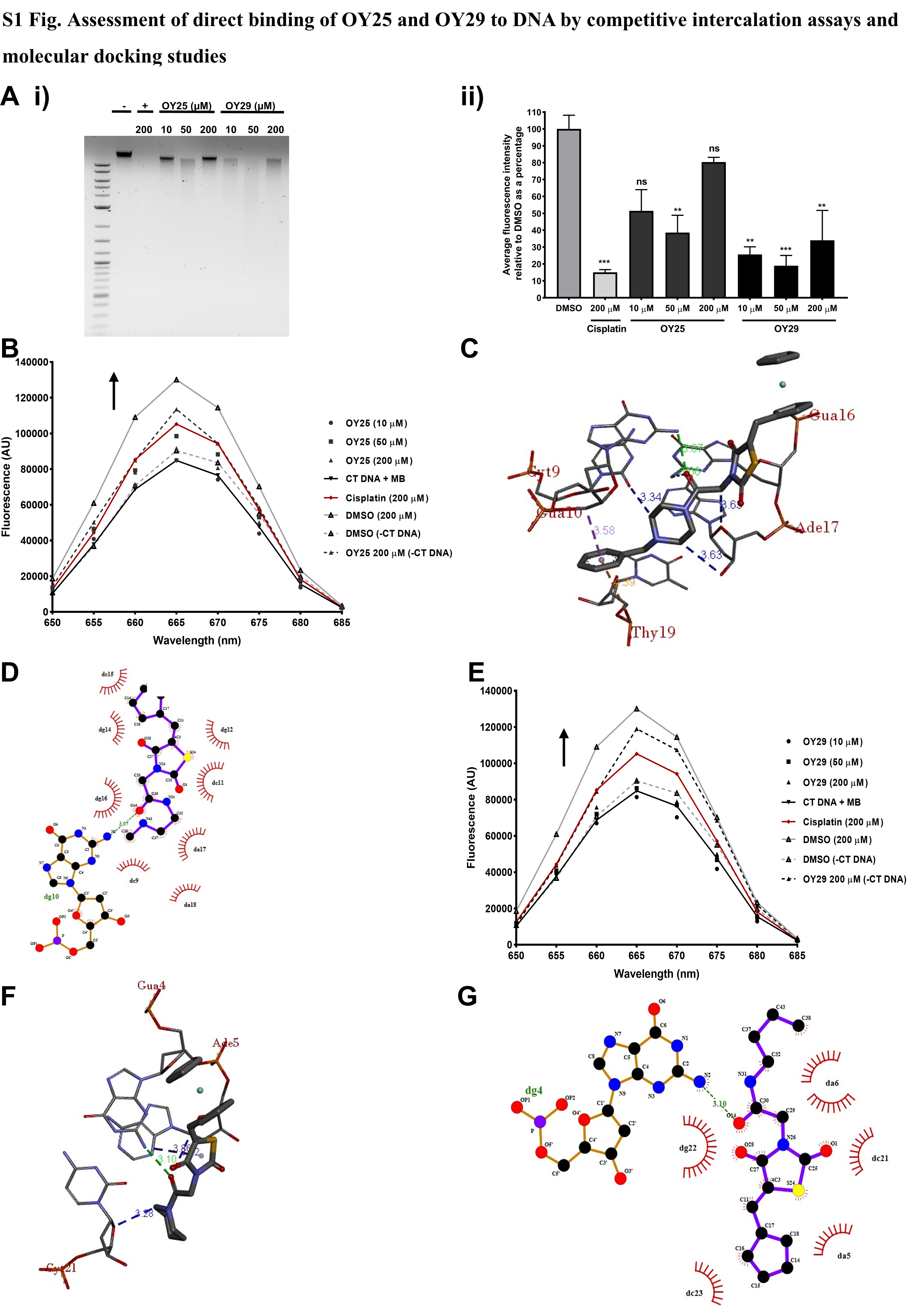

Supplement: S1 Fig — (A) Ethidium bromide competition for assessment of interaction of OY25 and OY29 with human genomic DNA. (Ai) 0.8% (w/v) ethidium bromide-stained agarose gel containing DNA extracted from HCC70 breast cancer cells. + : cisplatin). (Aii) The average fluorescence intensity (n = 3, with SEM), representative of three biological replicates of the ethidium bromide staining of the DNA bands quantified using ImageJ and shown relative to the intensity of the DMSO control. Statistical analyis was carried out using One-way ANOVA with Bonferroni’s multiple comparisons tests, where ns = non-significant, **p < 0.01 and ***p < 0.001. (B and E) Fluorescence emission spectra (650–685 nm) of 100 ng calf thymus DNA (CT-DNA) in the presence of (B) OY25 and (E) OY29 at concentration of 10, 50 and 200 µM measuring competition with 15 µg/mL methylene blue for the ability to intercalate into DNA. Cisplatin was used as a positive control. Molecular docking of (C and D) OY25 and (F–G) OY29 to DNA (PDB: 129D). (C and F) DNA-ligand interaction of OY25 and OY29, respectively bound to DNA. (C) OY25 and (F) OY29 DNA binding site containing interacting residues in maroon, hydrogen bonds in green dashed lines and other non-bonding interaction/hydrophobic forces shown in either blue or purple dashed lines. Ligplot+ interaction map of docked (D) OY25 and (G) OY29 in the DNA binding site. (D and G) The ligands (OY25/OY29) are shown as ball and stick projections with the black balls representing carbon atoms, the purple line representing the bonds between connected atoms and the brown line representing the base interacting with the potential hydrogen bond formed. Red, blue, purple and yellow balls represent oxygen, nitrogen, phosphate and sulfur, respectively. Hydrogen bonds are shown as green dashed lines together with a bond length/distance in Angstrom (Å). The red bristle represents the non-ligand (bonding) interactions/residues (DNA) involved in hydrophobic interactions of the docked compounds (OY25 [file pone.0328155.s002.tif]

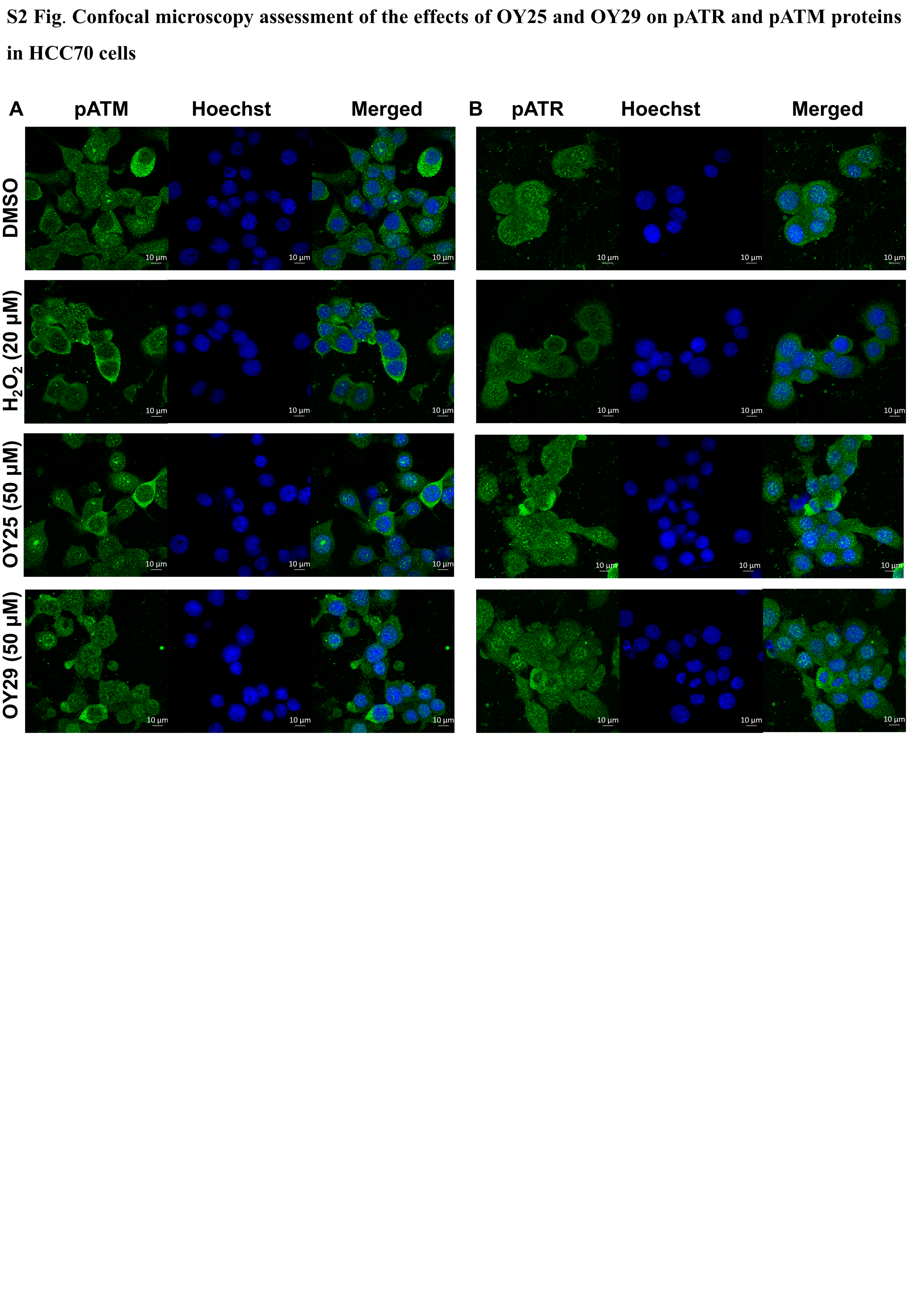

Supplement: S2 Fig — HCC70 cells were treated with 0.05% (v/v) DMSO vehicle control, 20 µM H2O2, 50 µM OY25 or OY29 for 2 hours. A-B Cells stained for (A) pATM and (B) pATR visualized under a Zeiss LSM780 Meta Confocal Microscope at λex = 488 nm and λem = 562 nm laser and images taken (n ≥ 30, cells) using Zen Blue software (Zeiss). Green staining represents A) pATM and B) pATR with the nucleus shown in blue, following staining with Hoechst 33342. (TIF) [file pone.0328155.s004.tif]

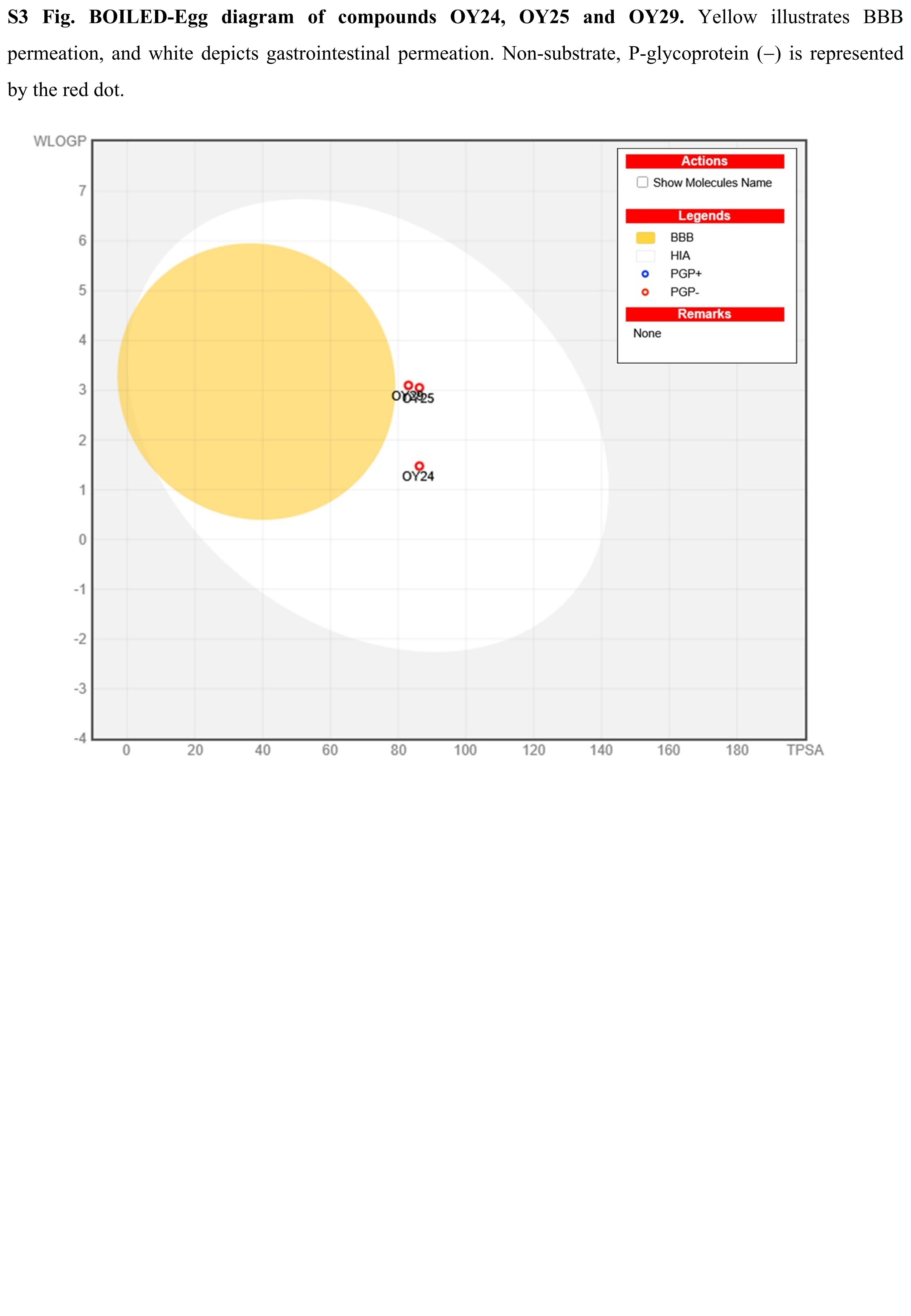

Supplement: S3 Fig — Yellow illustrates BBB permeation, and white depicts gastrointestinal permeation. Non-substrate, P-glycoprotein (−) is represented by the red dot. (TIF) [file pone.0328155.s006.tif]
