# Supplementary material for: Ferrocene thiazolidine-2,4-dione derivatives cause DNA damage and interfere with DNA repair in triple-negative breast cancer cells
Source: PLoS One. 2025 Jul 17;20(7):e0328155. doi: 10.1371/journal.pone.0328155 (PMC12270111; doi:10.1371/journal.pone.0328155)
Supplement: S2 Table — (DOCX) [file pone.0328155.s003.docx]

**S2 Table: Binding affinity and molecular interaction of OY25 and OY29 with DNA (PDB:129D).**

| **Compound** | **Binding affinity**  **(kcal/mol)** | **Hydrogen bonds** | **Distance of hydrogen bonds (Å)** | **Hydrophobic force (non-ligand residues)** |
| --- | --- | --- | --- | --- |
| OY25 | -8.4 | Guanine 10 | 3.07 | Cytosine 9,11  Guanine 12,14,16  Adenine 17, 18 |
| OY29 | -8.1 | Guanine 4 | 3.10 | Adenine 5,6  Cytosine 21,23  Guanine 22 |
