# Supplementary material for: Ferrocene thiazolidine-2,4-dione derivatives cause DNA damage and interfere with DNA repair in triple-negative breast cancer cells
Source: PLoS One. 2025 Jul 17;20(7):e0328155. doi: 10.1371/journal.pone.0328155 (PMC12270111; doi:10.1371/journal.pone.0328155)
Supplement: S3 Table — (DOCX) [file pone.0328155.s005.docx]

**S3 Table. Predicted physicochemical properties and some drug-likeness parameters of ferrocenyl thiazolidine-2,4-diones OY24, OY25 and OY29.**

| **Compound** | **Mw/g.mol^-1^** | **XlogP3** | **#HBD** | **#HBA** | **#NRB** | **TPSA/**Å^2^ | **PA** |
| --- | --- | --- | --- | --- | --- | --- | --- |
| **OY24** | 453.34 | 2.45 | 0 | 4 | 6 | 86.23 | 0 |
| **OY25** | 515.40 | 4.23 | 0 | 3 | 7 | 86.23 | 0 |
| **OY29** | 438.32 | 3.49 | 0 | 3 | 6 | 82.99 | 0 |

**Note:** Molecular weight (MW), atomistic octanol-water partition coefficient (XLogP3), number of hydrogen donors (#HBD), number of hydrogen acceptors (#HBA), number of rotatable bonds (#NRB), topological polar surface area (TPSA), PAINS alert (PA).
